# Determining capacity and barriers to disability sport development: the international federation of cerebral palsy football membership perspective

**DOI:** 10.3389/fspor.2026.1773783

**Published:** 2026-05-28

**Authors:** S. G. Arthur-Banning, Y. S. Oh, M. Domka, M. Fernandez

**Affiliations:** 1 Clemson University Department of Parks, Recreation and Tourism Management, Clemson, SC, United States; 2 School of Management and Strategy, Hong Kong Metropolitan University, Hong Kong, Hong Kong SAR, China; 3 The US Center for Mental Health and Sport, Clemson, SC, United States; 4 College of Applied Health Sciences, Justice, Equity, Diversity, Accessibility, and Inclusion, University of Illinois Urbana-Champaign, Urbana-Champaign, IL, United States

**Keywords:** CP-Football, disability sport, IFCPF, non-profit and voluntary sport organization, organizational capacity

## Abstract

Previously managed by the Cerebral Palsy International Sport and Recreation Association, Cerebral Palsy (CP)-Football is a para-sport for individuals with neurological impairments such as cerebral palsy, stroke, traumatic brain injury or similar neurological impairments. The sport is currently governed by the International Federation of Cerebral Palsy Football (IFCPF), a non-profit sport organization based in the Netherlands. Since the first Paralympic event CP football took part in, the 1984 New York Paralympic Games, which had 6 countries (Belgium, Canada, Great Britain, Ireland, Portugal, and United States) competing for gold, CP-Football has significantly grown with 70 countries from African, Americas, Asian, European, and Oceania regions registered as members of IFCPF. As the world-governing body of CP-Football, IFCPF has been organizing international level events (i.e., World Cup, World Championships, Regional Championships, and U-19 Championships) to allow member organizations to experience high-quality football in world class venues all around the world. In addition, the International Paralympic Committee, coupled with the various National Paralympic Committees have also been supportive of national level tournaments for the development of the sport. Even with its long history and the support from many sport organizations toward growth, several member organizations of the IFCPC are struggling to grow the sport in their countries, and at the same time, compete in international tournaments. The IFCPF are also seeking to continue to develop their women's game which is needed for gender equality and growing participation around the world. Thus, the purpose of this study is to identify the barriers to and facilitators of growing the sport of CP-Football in IFCPF member organization countries that are in their developmental stages of establishing the sport. In addition, this study seeks to determine the barriers to and facilitators of their participation in international CP-Football events. This study is framed based on an emerging conceptual model within the non-profit and voluntary organization literature, and the organizational capacity framework of Hall et al., used to examine the current issues faced by the IFCPC member organizations around the world.

## 1 Theoretical framework and literature review

### 1.1 Organizational capacity in non-profit and voluntary sport organizations

Non-profit and voluntary sport organizations (NVSOs) account for about 20%–25% of all non-profit and voluntary organizations across countries (1–3). NVSO is defined as an organization that primarily focuses on offering sport, recreational, and physical activities to make a difference in the quality of life within a community (4). In general, non-profit and voluntary organizations are structured, non-governmental, self-governing, non-profit-distributing, and voluntary in some meaningful extent (5). These organizations play a vital role in society; however, the contributions and efforts they offer have received little attention. Furthermore, research on the challenges they face in fulfilling their organizational missions remains scarce. Thus, it is imperative to seek to study various capacities within these organizations and try to understand their barriers (6).

Hall et al. (7) introduced a conceptual model of organizational capacity in the context of non-profit and voluntary organizations. This framework has been of interest in sport literature with scholars applying it in the case of NVSOs to explore the strengths and challenges they face in meeting their organizational goals (8–12). Within the NVSOs, organizational capacity is understood as the ability of an organization to use its internal and external resources (i.e., human, financial, and structural capacity) to achieve its mission of providing sport and recreational opportunities (9, 13, 14). In other words, it is conceptualized as a multidimensional model, which includes a set of organizational attributes that are believed to be essential in helping NVSOs accomplish their goals and meet their stakeholders’ expectations (15, 16).

### 1.2 The various capacities in non-profit and voluntary sport organizations

According to Hall et al. (7), non-profit and voluntary organizations depend on three types of capacities, which include financial, human resources, and structural capacity to fulfill their mission and objectives. Each capacity can be described more fully by first explaining the human resources capacity as it is the key dimension in shaping the development of the other two foundational capacities (7, 10).

#### 1.2.1 Human resources capacity

This refers to “the ability to deploy human capital (i.e., paid staff and volunteers) within the organization, and the competencies, knowledge, attitudes, motivation, and behaviors of these people” [Hall et al. (7), p. 5]. Among the two examples of human capital, volunteers are indispensable in operating non-profit and voluntary organizations as most of the organizational work is performed by them (9, 14, 17–19). These volunteers include coaches, trainers, officials, administrators, and committee members in the NVSOs, and most of the volunteers oversee multiple roles (20). Generally, NVSOs rely heavily on volunteers since they require large numbers of individuals to deliver sport services and have limited resources both financially and in their capacity to serve the games (21). Therefore, it is crucial to recruit volunteers who are committed and enthusiastic, while focusing on retaining sufficient numbers to support human resources capacity in NVSOs (4, 8).

#### 1.2.2 Financial capacity

This refers to “the ability to develop and deploy financial capital (i.e., the revenues, expenses, assets, and liabilities) of the organization” [Hall et al., (7), p. 5]. According to previous scholars, revenue sources of NVSOs include membership fees, sponsorship, fund-raising, public subsidy and government funding (8, 9, 16) in addition to a percentage of tournament revenue secured from a local organizing committee. Revenues can be distinguished between internal and external sources, where membership fees are categorized in the internal revenue, while the latter, such as fund-raising, viewed as external revenues (22). Most NVSOs consider membership fees as their primary source of income, which often accounts for two-thirds of all revenue sources (2, 23). However, revenue sources vary among NVSOs, where membership fees might be an alternate source for some even though potentially the most stable source of income. For instance, in the study of Lasby and Sperling (24), the authors examined sport and recreation organizations in Ontario, Canada, and found out that these NVSOs generate their main income revenues from corporate sponsorships, gifts, donations, and grants, which constituted 30% of all revenues, followed by membership fees. Revenue diversification is found to lead to greater financial stability for an organization (25). As stated by Coates et al. (22), sport clubs with a wealthier financial status tend to have fewer problems in volunteer management because they are able to provide greater support in training, and opportunity for their volunteers consequently allowing for increased retention. Thus, financial capacity can be said to be partially linked with human resources capacity, and that it is significant to have adequate funds to prevent other organizational issues from developing.

#### 1.2.3 Structural capacity

This refers to “the processes, practices, accumulated knowledge, and support structures within an organization that help it to function” [Hall et al., (7), p. 37]. Structural capacity is subdivided into three types of capacities, which include relationship and network capacity, infrastructure and process capacity, and planning and development capacity. *Relationship and network capacity* is the ability to develop and maintain relationships with the internal and external stakeholders (7). This capacity is a fundamental element alongside human resources capacity as it allows the NVSOs to get access to shared resources, exchange information and knowledge, and build social capital to develop the overall capacity (9, 14, 26). *Infrastructure and process capacity* is the ability to deploy infrastructure and internal resources such as databases, manuals, policies, and procedures to implement day-to-day operations (7). In NVSOs, formalized structure, good internal communication between members, and availability and quality of facilities are critical aspects of infrastructure and process capacity (8). *Planning and development capacity* is the ability to develop strategic planning, creative planning, and implementing the plan (7). According to Wicker and Breuer (11), strategic planning helps to reduce issues within an organization and will help to foster a positive impact on the development of a sport organization. Specifically, it is crucial for NVSOs to have strategic budget planning as it helps to prevent financial problems (27). Therefore, NVSOs relying heavily on external revenues might have a higher probability of facing financial issues due to the unprojectable external funds from public subsidies or government funding, which might change from year to year (22).

Overall, although human resources capacity is said to be the key factor of organizational capacity in non-profit and voluntary organizations, a good balance of all three capacities may greatly influence the ability of an organization to fulfill its mission and achieve its objectives (7, 14). However, it is difficult for an organization to maintain all the necessary resources to achieve its objectives, particularly in smaller organizations. Hence, they are obliged to depend on external resources to fulfill the scarcity of internal resources (28).

### 1.3 Challenges faced among non-profit and voluntary sport organizations

NVSOs continue to face challenges in a number of the capacity building areas, specifically struggling with an insufficient and/or shortage of volunteers (2, 3, 8, 9, 16, 21, 27, 29, 30), a lack of financial stability and management (2, 9, 16, 31), weak strategic planning initiatives or implementation of the plan (9), and infrastructural problems such as lack of internal communication and limited access to safe and/or suitable facilities (3, 9, 16, 31).

Compared to other non-profit and voluntary organizations, NVSOs are more likely to face human resource capacity problems as these organizations rely heavily on volunteers for everything from staffing to coaching and training (2, 9, 16). In addition, prior literature on NVSOs indicates human resources capacity was found to be most critical, especially around issues related to volunteers, since paid staff increased over time as organizations get more established and budgets are more secure (9, 16). To provide one specific example, Wicker et al. (31) in their study found that recruitment and retention of volunteers was the biggest challenge among sport clubs in Germany and Switzerland. Furthermore, recruiting and retaining the right type of volunteers needed was also found to be a critical issue (2). Yet, volunteer management practices are receiving scant attention from these sport bodies (14, 16, 27, 32), and there seems to be a decline in volunteers in the NVSOs (14, 20, 30, 33). This is often due to the lack of free time among volunteers, coupled with the lack of resources among NVSOs, leading them to face challenges in volunteer recruitment and retention that are not easily addressed (3). In addition, volunteers are given multiple roles, often feeling burdened by the increased level of responsibilities put upon them (9, 12, 14, 32).

Along with the human resources capacity, financial capacity was also found to be a challenge for NVSOs due to a substantial reduction in public subsidies and funding opportunities (7, 2). This decrease in financial support often forces NVSOs to search for alternate funding sources to fulfill their goals (33). However, it is unfortunate that NVSOs tend to have fewer financial resources as compared to other non-profit organizations as sport is seen to be revenue generating without the recognition that only in the professional realm of sport is revenue generated and thus the misconception that all sport organizations are cash rich (2, 18, 24). In addition, Lasby and Sperling (24) revealed that NVSOs often report difficulties in earning income, obtaining funding from the government entities, individual donors, and other revenue sources, again likely as a result of the misperception that sport generates revenue. Consequently, even though human resources capacity was revealed as the most important dimension (31), financial capacity should not be ignored as securing stable funding is a critical component for the success of non-profit organizations (34).

In addition to funding and volunteer obstacles, challenges in the structural capacities faced by NVSOs are found to be associated with a number of different elements. For example, NVSO's often experience deficits in human resources capacity and financial capacity, yet often see issues related to relationship and network capacity, infrastructure and process capacity, and planning and development capacity, making it evident that many are interrelated with one another other (7, 9). Furthermore, in the study of Hall et al., (7), the authors found that an insufficient human resources capacity (i.e., volunteers and/or paid staff with strategic planning skills) and a lack of financial capacity (i.e., core funding and stable and/or long-term funding) restrict development of organizational strategic planning for many NVSOs. In terms of infrastructure and process capacity, Wicker and Breuer (16) revealed that this dimension is less severe as compared to human resources capacity and financial capacity but still one not to be ignored.

Previous scholars examining organizational capacity in sport have mainly focused on community sport organizations (8–14, 16, 27). Recently, studies on organizational capacity have gained more interest in the field of sport management, where the theory has broadened by covering sport for development and peace organizations as well (35–37). However, studies related to sport federations and their organizational capacity remain scarce. As Wing (38) noted, “Whatever capacity building might be, it is not going to be the same across such a diversity of kinds of [nonprofit] organizations” (p. 154). Thus, this study aims to fill the gap of organizational capacity literature by examining sport federations across different country members. It is crucial to understand the organizational capacity of different IFCPC member organizations as there have been few studies on this topic, and further examining the needs of the various member federations could potentially lead to greater sport development opportunities within a country and certainly within the IFCPF federation.

## 2 Methods

In order to address the study's research questions, the study employed a qualitative approach. Data was collected via participant interviews in a number of formats, including zoom, phone, and in person interviews.

### 2.1 Data collection procedures

The goal for recruitment was to obtain participation from coaches or administrators who were in the developmental stages of establishing or strengthening their Paralympic soccer programs as identified by the IFCPF leadership. The IFCPF identified 20 countries who were in the emergent stages of developing their Paralympic teams or countries who did not have an active program. A subsequent email was sent to those countries from the research team requesting their support in the research. In addition, the research team recruited country participation from an IFCPF qualifying tournament for the world Championship held in Europe. As a result, three programs representing Germany, Denmark, and Scotland were interviewed at the tournament.

Additional interviews were conducted after five coaches, and three administrators contacted the research team after the tournament. Out of those eight programs, the research team was unable to coordinate a conference call with one country. As a result, a total of ten programs (3 at the event and 7 follow-up calls) preliminarily agreed to participate in the study, but only 9 total programs were interviewed as receiving regular communication within the research timeline was difficult for one country. Interviewees represented were Austria, Kazakhastan, Wales, Chile, Scotland, Denmark, Germany, Nigeria and South Korea. In general, the participants representing their country's federation were mostly male (8 men, 1 female) and were mostly coaches.

### 2.2 Interview protocol

The interview questions for this study built off of the organizational capacity framework and the core research questions that examined: (a) facilitators, (b) constraints, and (c) recommendations to the IFCPF. The study utilized a semi-structured interview guide which included an opening script, key open ended questions guided by the theory, followed by additional questions to seek clarification, elaboration or further reflection to gain a better understanding for the participant responses (39). In order to create rapport, interviewees were asked about their role with CP-football and the CP-football infrastructure within their country. Interviewees were then asked about the organizational support their team receives in the form of leadership, training, and partnerships. Study participants were also asked to comment on the perceived benefits their team would gain through participating in international CP-football training or events. Further questions were asked regarding the national policies in place that promoted or constrained participation in international tournaments. Finally, participants were asked for recommendations for the IFCPF to increase international participation in CP football among developing and/or smaller countries. As such, the following questions in Table 1 were developed for interviewing study participants.

**Table 1:** Interview questions.

Categories	Questions
Interview background	Can you tell us a bit about you and your background/history with CP-football?
	Can you tell us about your organizational structure and the structure of CP-football within your country?
	What tournaments or events are you aware of that the CP-football team has competed in recently?
Organizational support	What resources are available to your organization for participating in CP-football?
	What are the priorities of the leadership of your organization?
	Are there any aspects of (your) (the) leadership that will help grow participation? If so, can you tell me more about it?
	Are there any aspects of (your) (the) leadership that might hurt growth of participation? If so, can you tell me more about it?
	Can you tell me more about the training resources the organization has access to that helps with growing CP-football in your country?
	Does your organization work with other soccer associations?
	How do partnerships help or hurt your organization in participating in the CP-football?
	What rewards/benefits does/would your organization receive from taking part in the International CP-football?
Policies	Can you tell me more about the any policies in place that help or hurt your organization from participating in International CP-Football?
Recommendation	How can the IFCPF help your organization to grow the sport of CP-football in your country?
	How can the IFCPF help your country compete in more international tournaments?

Three of the interviews were conducted face-to-face, and four were conducted through Zoom. The coaches from Nigeria and South Korea requested the interview protocol and answered the questions in a word document; therefor, the document was emailed back to the research team so follow-up questions were difficult to obtain for these two countries. Interviews conducted face-to-face or through Zoom were audio recorded with the permission of participants. The research team conducted the interviews, and two bilingual researchers, who speak English and Spanish, and English and Korean, were present for the programs where these languages would be of assistance. The coach from Kazakhstan utilized his own interpreter for the interview that was present on the phone with the coach as well. Interviews ranged from 32 to 50 min, with an average time of 42 min.

### 2.3 Data analysis

Interviews were recorded and transcribed verbatim using a transcription service. Each study participant received a pseudonym during the transcription process to protect their confidentiality. An inductive approach was then used to identify emerging themes in participants’ narratives (39). Emerging themes were identified using open coding in participants’ narratives and further assigning of themes. Similar themes were then grouped and merged when applicable. More specific thematic areas addressing the study's research questions were then identified.

Upon identification of the themes, participant confirmation was conducted by returning interview transcripts and emerging themes to participants to ensure accuracy and enhance credibility (39). Information was sent back to the participants as a form of member checking and confirmation that information provided in the interview sessions was consistent with the themes that emerged as a result of the data analysis. While participant response was often vague, each confirmed that even if a theme was identified that was not specifically stated by that individual participant, it certainly was a theme they were experiencing in some capacity and verified its usefulness.

## 3 Results

There were three major themes that were identified in participants’ narratives. The first theme, “Threats,” summarized internal and external threats organizations faced while creating or strengthening their teams. The second theme “Organizational Assets,” summarized the perceived internal factors within the respective organizations that coaches and administrators felt were conducive to creating or strengthening their teams. The third theme, “Recommendations for growth,” discussed items that the countries believe the IFCPF could provide support to that would help the respective countries create or strengthen their Paralympic soccer programs.

### 3.1 Threats

#### 3.1.1 External threats

Various external threats were mentioned by participants. Among these types of threats, coaches shared stories related to underdeveloped infrastructure and few resources, politics, and stigma associated with the population they serve.

##### 3.1.1.1 Underdeveloped infrastructure and few resources

Several coaches struggled with their teams due to their country's infrastructure. The coach from Country 1 stated, “It's pretty bad in our country at the moment because we don't really have any structure. We really need to work on it. There aren't any clubs.” The coaches from Country 2 and Country 3 further echoed these sentiments. In the case of Country 2, the coach mentioned not having funding from the government. In the case of Country 3, the coach added that the country was:

…not a country rich in personal resources such as Brazil or as the US, [or as] England, as Germany, as Ukraine, as Russia- where you just have a huge population- where you have more possibilities of finding people and resources. We are a small country.

The coach from Country 4 shared the following:

The nature of the country, the population is very much spread out, apart from the city. In the south, for example, our capital has 350,000 people. That's the biggest populated area in the country. Then, the population is spread out, so you might have…In the middle of the country, for example, is probably 80% of our landmass, yet there's only 6% of the population live within that area. Population is an issue, in terms of having a critical mass to create a national team, and that's been one of the issues that we've had.

Other infrastructure challenges dealt with visas and identifying eligible players. For instance, the coach from Country 2 wrote,

Visa policy here is very complicating just like what happened when CPFAN applied for Visa with Spanish Embassy to participate in the 10th Barcelona International Trophy of CP-football held in June 2015; all applicants were refused, and we could not participate in the competition.

The coach from Country 4 further mentioned that there was no way to identify the players with a disability due to confidentiality issues. In addition, the administrator from Country 5 added, “We face difficulties in finding new talented players. Even if we found new players, they will end up switching their sport to individual sporting events such as track and field or bocce.”

##### 3.1.1.2 Politics

Politics could also affect the support that a team receives. In most cases, while it is recognized that the political climate in an individual country may be difficult to generalize, it is still of vital importance for an organization to understand the different political situations that programs are facing. For example, the coach from Country 3 shared the following story related to the politics in his country:

Then, we also had a bit of a shock with our sports ministry. There was a big scandal in our sports ministry, where there was some kind of money laundering going on. They cut all of the federation’s funding, not only disability sports federation, but also all sports federations. One of the cuts in the budget, one of the sports that were affected was us. That’s why we were not able to make it to Denmark this year to the developing tournament.

##### 3.1.1.3 Stigma associated with the population they serve

Among the challenges coaches faced, stigma associated with people with disabilities was a concern. At times this meant that individuals with disabilities hesitated to be involved with causes or programs that highlighted their status as having a disability. Other times, individuals with disabilities were constantly treated so that they were not capable of participation. For example, the coach from Coach 3 shared the following:

They spend their lives trying not to be disabled and trying not to have any involvement with disabilities if they even know that they have a disability. On the other hand, those ones that have more severe impairment are thrown into a system which tells them they are not capable of doing anything, that they have a mental disability, or are intellectually challenged.

The coach from Country 6 echoed a similar sentiment. He stated,

If people are on the certain stage, they don't want to get in touch with…all this. For me it was (or it is still) the big goal to get normal. To still get identified; to just walk through the city…I think that's the point because no one wants…to have a disability.

The coach from Country 3 added that in some countries individuals with CP will most likely be “from poorer backgrounds” with little to no access to sports.

#### 3.1.2 Internal threats

In addition to external threats, the participants mentioned several internal threats within their agencies that could hinder creating or strengthening their Paralympic teams. Among these types of threats, coaches shared stories related to attrition and retention, and lack of finances.

##### 3.1.2.1 Attrition and retention

Attrition and the inability to retain players was a challenge mentioned by coaches. The coach from Country 4 mentioned that he currently had a small team composed of approximately 10 players. He shared that most of the players dropped out of the team because of family or work matters. He also shared the following story related to the effects strong players had on the team:

Any time we get a new player (and it’s not often, but it’s like once or twice a year maybe), the player comes once and if one of the good players is missing (one of the three or four good players are missing, which always happens if somebody’s missing due to whatever reason), the player’s not really going to come back. He’s going to see a lot of disability, and he’s going to leave. We have a hard time keeping very strong players. On the other hand, weaker players will come. They see the top competition because even our weak players, they've been practicing already for a couple of years, so they have a big advantage over new players. They don't want to stay either. We've been, over the last year and a half, having troubles getting players to stay with our program.

##### 3.1.2.2 Lack of finances

Coaches also mentioned lack of finances as a common challenge. The coach from Country 4 stated, “Being completely honest with you, we don't have a massive amount of money, nowhere near the sums that some of the other governing bodies can throw at things.” The coach from Country 7 also stated, “We only have the resources of a … Paralympic Committee, and we are trying to develop our federation of CP-Football, but it's very difficult because we don't have a lot of support of the people who are working in CP-Football. It's very difficult, but we are trying to make this federation have more resource to have more development.” When asked to clarify what resources he was referring to, the coach mentioned that he was referring to funds as they were only provided with a “field and some infrastructure.” The coach from Country 4 mentioned having enough funds to run his grass-roots level team. However, his funds were not enough to host an international team.

#### 3.1.3 Organizational assets

Although coaches mentioned various threats to creating or strengthening their teams, several organizational assets were mentioned by participants. These types of organizational assets included current leadership and partnerships.

##### 3.1.3.1 Leadership

These coaches in the study reported significant experience with their teams. Multiple coaches were involved in their national team coaching programs with top certifications and licensures demonstrating that the content knowledge and leadership they can bring to the program was evident. The coach from Country 1 stated the following, “I have experience in working with national teams in my country. I have an ‘A’ category license.” The coach from Country 2 also reported experience with successfully organizing “a coaching education workshop in 2015 with two foreign instructors present, a Super 4 tournament in 2016, and a trial for selecting our National team in October of 2019.”

##### 3.1.3.2 Partnerships

Partnerships were also mentioned throughout participants’ accounts. The coach from Country 2, for example, wrote the following: “The Paralympic Committee… helps CP-Football in the usage of football pitch during tournaments. [The committee] also helped in the recommendation of CP-Football to other organizations.” The coach from Country 8 added that his partners were “giving me fields, inventory for the trainings, and they're paying me as well. Yeah, a full help.”

The coach from Country 3 shared the following of a local football club:

…they invited us for training camp and they liked us. We had a friendly game in front of like two-hundred people or something. They said we should do a tournament. The opening game of this first tournament that we did in 2018, we had five-hundred spectators or something on the pitch. It was a very, very big event. Nowadays, it is like one of the main events of the season when all the CP players come down and play and then party and everything. Then, we get a lot of local support from there, financial support as well. Hotels, they invite us, we get the pitch. We get pretty much everything financed. We are there for free and we get to get all the other teams for very, very good conditions. That's been pretty much how we've been affording to get international competition down here. Teams will come; we don't have to pay. It all works out very nice. People are very friendly there. It's been very good, so far.

The coach from Country 4 further mentioned having built a group of partners. He stated that the group “meet up on a yearly basis and just hashed out what will happen in terms of the grassroots side of things, coached the international side, and then just try and make sure that we're all singing off the same hymn sheet.” In addition, the chair from Country 5 mentioned “We work with the Paralympic Committee and the Paralympic Football Association to grow the sport of CP-Football in the country. We also have a great relationship with the Japanese CP-Football Association and Japanese CP-Football Teams and have been initiating CP-Football Exchange Games for nearly 20 years. This helped our players to improve their football skills.”

The coach from Country 7 further mentioned that he closely worked with their Paralympic Committee and the national federation of football. The coach from Country 6 also worked closely with the Disability Association. However, although the countries had working partnerships, they reported that there was room for growth.

#### 3.1.4 Recommendations for growth

All the coaches had recommendations to share regarding how the Federation could help them create or strengthening their teams. The most important and prominent themes that emerged were hosting education and training, and increasing financial support.

##### 3.1.4.1 Education and training

Several coaches recommended education sessions or training in order to create or improve their teams. The coach from Country 1 asked for “a seminar for coaches of football.” He added, “We really need some books or other materials of how to teach…That would really help us.” Similarly, the chair from Country 5 added, “There are no professionally trained CP coaches in our country, which leads to lack of skill development for players.” On the other hand, the coach from Country 3 mentioned that he did not need help to instruct players how to play football. Rather, “the most important part of this is teaching coaches how they recognize CP players. What we're looking at as coaches who are starting to need to know when they see a CP player, like this is the guy that you're looking for.” The coach from Country 6 added that his team needed information related to the classification system because “we didn't know anything. The athletics classification is completely different. That's why they get all the classified athletes and football, we are beginners.” The coach from Country 3 also offered to host workshops related to the classification system.

Education was also related to creating awareness of CP and increasing publicity. The coach from Country 2 wrote, “I will like to add my suggestion that IFCPF should appoint two/three persons from the registered country members to create awareness (face to face advertisement) and visit neighboring countries for participation in CP-Football.” He also asked for IFCPF to “provide adequate information to [the] embassy/home affairs office where competitions will hold to ease the issuance of entry visas” and “work more on publicity so that the organization will be recognized WORLDWIDE by governments, embassies, companies, schools, etc.”

##### 3.1.4.2 Financial resources

Coaches from various countries also mentioned that they would benefit from financial resources to either strengthen their teams or be able to afford participation in international tournaments. The coach of Country 3 mentioned, “One thing that would make things easier is for official tournaments to be more affordable.” In order to do this, he suggested that the format of the tournament could be changed to take over a span of about 4 days instead of 2 weeks. The coach from Country 2 also wrote that “IFCPF should get more sponsors so that participating fees for tournaments will be reduce for example just like the just concluded. IFCPF World Championships Qualification Tournament [was] very expensive for us to participate.” In his case, his team could have also benefited from IFCPF “providing more facilitating equipment/kits for players.” He finalized by recommending that the organization take into “consideration countries who have foreign exchange inflation while fixing registration fees for participation in competitions.” The coach from Country 3 advised for more local tournaments that could be more affordable.

## 4 Discussion

**Figure 1:**
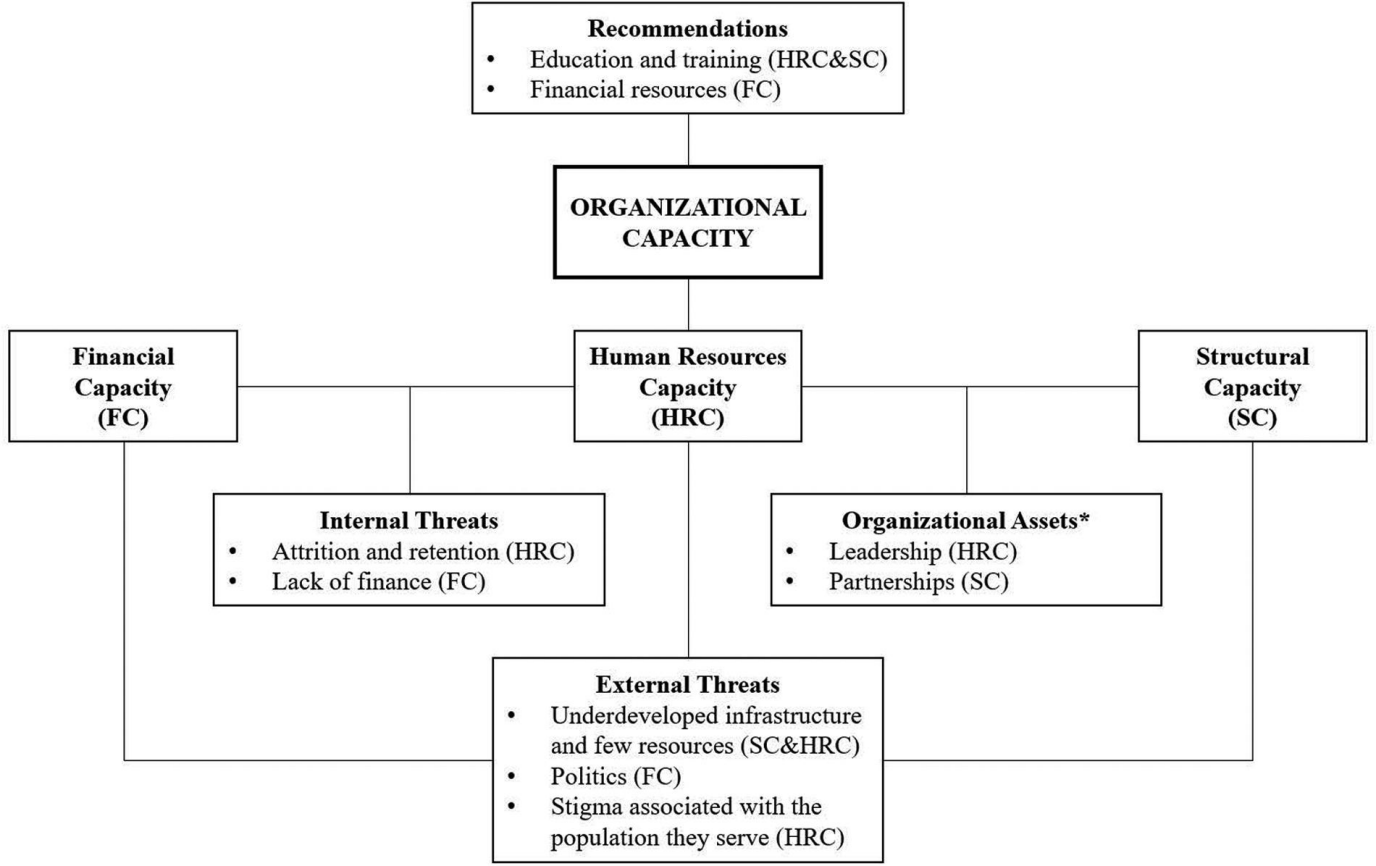
Overview of organizational capacity model in IFCPF member organizations.

The aim of this study was to gain insight into the challenges faced by an international Paralympic sport federation membership, specifically, the IFCPF member organizations in terms of organizational capacity in growing the sport of CP-Football in their respective countries. Furthermore, to examine the barriers to and facilitators of participating in IFCPF tournaments. Human resources capacity and financial capacity were most mentioned by the participants as the biggest challenge, while structural capacity was found to be less severe among the participants. Figure 1 highlights the interrelationship overview of our findings with the structural elements guided by from Hall et al. (2) Nonprofit capacity model.

### 4.1 Human resources capacity

Human resources capacity was found to be associated with all the themes identified in our findings. Unlike NVSOs in general, where volunteer recruitment and retention were the critical issues (2, 9, 16, 31), member organizations of the IFCPF are struggling in finding and/or identifying athletes (external threat) who are eligible to participate in CP-Football. Some of them stated that the nature of the country, where they only have a smaller population as compared to other countries, is restricting them from finding potential athletes. On the one hand, issues around athlete recruitment might be due to the negative attitudes toward people with disabilities from society (4, 41), which decreases the willingness of athletes to fully engage with public sports programs. However, studies have also indicated that people with disabilities often receive a negative portrayal from the media (42, 43), or their participation is seen as a charity opportunity rather than an opportunity to be competitive in sport. Thus, one of the challenges that has emerged seems to indicate that people with disabilities tend to hide their disability due to the stigma of being “disabled” (external threat), which further denies them any disability related activities or potential services. This, as a result, puts coaches in a difficult situation in recruiting their athletes but also encouraging the athletes to be proud of who they are and acknowledge their disability as empowering to the Paralympic movement. However, findings related to stigma might differ in future studies as our results are based on coaches’ or administrators’ perspectives and not athlete's perspectives specifically.

In addition, difficulties in recruiting and/or identifying athletes tend to combine with lack of having professionally trained CP coaches (external threat), which was mentioned as one of the challenges by the participants. This is perhaps not surprising as previous studies have already pointed out lack of qualified coaches as an issue when offering sporting activities for people with disabilities (41, 44, 45). Based on our findings, professionally trained coaches are those who have the skill set of coaching individuals with disabilities, as well as having knowledge of the specific sport itself. Therefore, it can be difficult for the coaches to identify potential athletes, and at the same time, deliver appropriate training sessions for the athletes if they don't have the knowledge of the disability or in the case of the IFCPF, multiple disabilities as the sport encompasses individuals with Cerebral Palsy, individuals who have experienced traumatic brain injuries, or stroke which all present and often have different disability programming needs.

Communication with programs that are more developed or clinics hosted by experienced coaches could help support some of the barriers that the countries interviewed are facing. Sharing experiences from a variety of countries can contribute to more communication and positive growth across the board. Perhaps a coaches committee could become a more formal structure within the IFCPF organization to increase capacity.

Another challenge that was found in the human resources capacity was related to attrition and retention of athletes (internal threat) (46). Our results revealed that athletes tend to drop out of the sport due to family or work matters, coupled with the absence of talented peers in the team, or they get recruited to other sport teams with a perceived more clear classification system like athletics. This study might be one of only a few to identify the challenge of elite athletes with disabilities dropping out from sport or switching sports, as previous studies have only looked at the barriers to and facilitators of sport participation among this population (41, 44). Particularly for a period when the IFCPF was first notified that they would not be involved in the Paralympic games in Tokyo 2020, and subsequently 2024 in Paris and 2028 in Los Angeles, this created a big change in Paralympic funding for a number of countries and also created a void where athletes who wanted to become Paralympians looked to change sports, so they could potentially make their Nation's Paralympic team. The IFCPF has just recently submitted a proposal to be included in the 2032 games in Brisbane, Australia and the hope is that this addition can change the funding structure of a number of the developing nations to more fully build out their CP Football sport program for both men and women. Interestingly, studies related to athlete dropouts have been implemented among the able-bodied population (47–50) and consequently, with a dearth of literature on athlete dropouts in disability sport, it is imperative to further explore the issue. As the IFCPF has been able to restructure their tournament hosting rights, it has increased their budget which is allowing more staff to be hired or contracted for services. While this does not directly impact the developing countries structurally, it does support them with lower tournament fees or increased opportunity through better channels of communication and more tournaments in at the regional levels which further encourages participation and builds capacity.

As human resources capacity was found to be the greatest challenge alongside financial capacity, result revealed that leadership (organizational asset), which lies within the human resources dimension was mentioned as one of the strengths by the member organizations. This indicates that the organizations have the skill sets to grow their federations and the sport but lack the resources to implement the ideas or programs. As stated by Pfeffer and Salancik (28), “the key to organizational survival is the ability to acquire and maintain resources” (p. 2).

### 4.2 Financial capacity

Most member organizations of the IFCPF stated a lack of financial support (internal threat) as a challenge in the development of their federations. This issue is similar to the study of Mojtahedi and Katsui (51), where funding limitations was one reason that was found to have a negative influence on the development of wheelchair basketball in Ethiopia. The authors stated that low financial support resulted in the low success rate in regular training, and as a result, negatively affected the chances of achieving growth in the sport. Furthermore, Brown and Pappous (52) in their study of NVSOs revealed that acquiring funds for the national disability sport organizations is a challenge as the organization needs to compete with other NVSOs for the same funds. Particularly if the team is not successful at international events, requesting funds from their national federations are difficult. This in turn perpetuates the cycle of low participation or less opportunity to train and improve, leading to poor performance at events and the cycle continues. This is common in the context of disability sport as there are multiple sports for people with disabilities in their initial stage with limited number of funds available. Just recently, as a result of this challenge, the IFCPF have taken greater control of a number of their international tournaments and have been able to secure funds for more affordable events or allows some of the programs more latitude in their participation.

Furthermore, political issues (external threat), which were categorized in the financial dimension were also found to have a detrimental effect on the member organizations. This finding is consistent with the study of Brown and Pappous (52), which suggests that national disability sport organizations will be greatly impacted during periods of austerity. Austerity is defined as “a form of voluntary deflation in which the economy adjusts through the reduction of wages, prices, and public spending to restore competitiveness, which is (supposedly) best achieved by cutting the state's budget, debts, and deficits” (Blyth, 53, p. 2). According to the author, people with disabilities will often experience a substantial impact on budget cuts during austerity as this is seen as an easy cut to make due to the low numbers that tend to participate in disability sport. Thus, for those NVSOs who rely heavily on public funds, it will have the greatest impact when it comes to political issues. In addition, it is recognized that while not an entire country austerity measure was put in place in any one of the countries involved in the research, by having the sport of CP-football removed from the Paralympic games in Tokyo, Paris and Los Angeles, this has caused a microcosm of funds being shifted to sports that were going to be in the Paralympic program and thus created cuts and reductions to CP programs much like an austerity measure might be reflected.

Financial support plays a vital role in sport development and for the success of the growth of the sport worldwide. In the study of Mojtahedi and Katsui (51), the authors gave an example with a case of people with disabilities in Mozambique to provide evidence on how external funds could help grow sport opportunities for people with disabilities. To be more specific, Mozambique, a southern African country, was able to create the Paralympic Committee of Mozambique, and further formed a Paralympic team to participate in the 2012 London Paralympics with financial support provided by The Abilis Foundation. However, unlike the successful case of Mozambique, there are an infinite number of countries in the NVSOs who lack financial support from a Foundation or other alternative funding sources and many of the countries looked to the IFCPF to help with such a program. Perhaps this could mean the IFCPF governing body could provide a template of sorts that a country could use to then add their own specifics to seek external funds from foundations or business. This would show the member countries that the IFCPF recognized the challenges faced by the membership and they are seeking to provide support in overcoming the barriers they face.

In addition, various clinics or trainings could be provided from the IFCPF around capacity building for not-for-profits such as fundraising efforts or initiatives to collectively communicate with other governing bodies like FIFA through various Football Associations to encourage more funding for the disability disciplines of the sport.

### 4.3 Structural capacity

Structural capacity was found to be the least important element as compared to the two capacities aforementioned. Among the three dimensions of structural capacity, infrastructure and process capacity was the only challenge mentioned by the member organizations. Having no structure, no clubs to compete, and visa related items (external threats) were the only three challenges revealed in this dimension. At least in the upcoming International events, the IFCPF is seeking to work with country governments to support visa applications and timely travel processes.

Relationship and network capacity was found to be another asset alongside leadership in the human resources dimension for most member organizations. Acquiring the relationship and network capacity allowed them to fill the absence of physical infrastructure (i.e., sport facilities), enhance athlete skills by participating in training camps and share training skills, and get financial support from the partners. Partnership with other NVSOs and/or for-profit organizations (i.e., community institutions) were essential for disability sport federations as it allowed them to decrease their dependency on government funds. However, according to the resource dependence theory introduced by Pfeffer and Salancik (28), organizations may lose their autonomy as partners who provide necessary resources may take control over them or seek to take them in a direction that may be different than the member country had in mind. In addition, given the rapid growth of the disability sport industry and infrastructure in some countries, a quick, significant power shift would be possible with the right circumstances and funding in place.

Surprisingly, planning and development capacity was not mentioned by any participants, which, according to the model, was said to be the most challenging among the three capacities in the structural capacity construct (7). This might be due to the lack of long-term stable funds from each member organizations, coupled with the absence of structures, where there are a few or no clubs to compete against. For this reason, the participants might not have a chance to be at the stage of planning, and thus only perceive the importance of human resources and financial capacities which are the more immediate issues for the member organizations to be addressed.

### 4.4 Recommendation for the international federation of CP-football

The specific needs that could help each member organization to fulfill their organizational capacities and to develop the sport of CP-Football in their respective countries were revealed. They are divided into two sub-themes: education and training, and financial resources. To be more specific, education and training is classified in the structural capacity, and the latter obviously falls under financial capacity. As previously mentioned in the human resources capacity section, there is a lack of expert professionals who can assist in training or have the knowledge to identify athletes who can play the sport within each country. Thus, to fulfill this gap, it is fundamental to secure a solid infrastructure and help provide more education to professionals or medical staff that work with individuals who work with people with CP, TBI or stroke so as to become stronger advocates for, or to provide referrals to, CP-football programs. While the IFCPF does have a training platform available free of charge for IFCPF members, that still may not be possible to get the grassroots medical professionals and programmers who work with potential athletes to take the courses. Similarly, the IFCPF could host a series of webinars about the sport targeted at National organizations or NPO's to further encourage education, identification and funding pathways for National teams to grow and compete.

Furthermore, providing the member organizations with the most up-to-date information on the classification system of the sport would be helpful in determining which athletes and populations to recruit most heavily. Providing CP-Football-related equipment for athletes and hosting more local tournaments were also advised by the participants. This helps cut costs for teams who may not be able to travel quite literally, halfway around the world to compete in an event but rather allows more regional type events with fewer costs and perhaps fewer teams so the tournament itself is shorter, each helping reduce costs while increasing tournament participation. In addition, visa issues were mentioned by member organizations, and it was suggested that IFCPF work with the various Local Organizing Committees to create more awareness within the country or to be keenly aware of where the events are hosted and the various political difficulties some countries might face by seeking to enter a country for participation during the tournament bidding process.

In terms of financial capacity, financial support was mentioned as a source of strengthening the teams and to participate in IFCPF events. Some participants suggested that the IFCPF change the tournament format by decreasing the event days so that it will be more affordable for the less wealthy programs to participate. While this might make the event more affordable, it also begins to put a strain on the athletes’ physical abilities as they will have less rest and recovery time between games, potentially affecting their performance. This is a tricky balance where greater research on rest and recovery of individuals with CP is needed.

Also, the member organizations recommended that the IFCPF seek to secure more sponsors so that all fees related to participation will be reduced significantly. With new administration within the IFCPF, this is become more of a reality. The IFCPF has begun to tap into organizations social responsibility funds to try to enhance awareness for athletes with disabilities but they are also raising awareness through social media to a point where it is enticing for companies to consider CP Football a great exposure opportunity. This information also needs to be communicated to the developing countries as opportunities for not only their participation but also their own sponsorship seeking.

## 5 Conclusion

This study, while exploratory, contributes to understanding the lack of each organizational capacity dimension of IFCPF member organizations. It is crucial to understand the challenges faced by each National federation as it allows the IFCPF to offer the missing elements for the development of CP-Football around the globe. In general, professional sport organizations require greater capacities to sustain and offer sport programs at the highest level to the athletes. Moreover, stakeholders are expecting professionalization from national sport federations, that is, a “transition from an amateur, volunteer-driven pastime to a more business-like sector” (54, p. 108). However, unlike sport in general, where they are supported by corporate sponsors with explosive media attention, disability sport federations have fewer resources available to accomplish their goals. Therefore, this study not only tried to help recognize the gap in the organizational capacity framework but also offers potential solutions to continue to build the organizational capacity in the disability sport federations. While it is fully recognized this is but one view into a specific sport organization, in framing the study in organizational capacity, it is clear that many of the challenges faced by the IFCPF member organizations, while not unique, perhaps provides opportunity for other sport organizations, particularly developing disability sport organizations to learn and grow from. It would be insightful to provide similar research questions to other disability organizations to determine if these same or similar challenges are faced by many of the other international disability sport federations in the future.

## Data Availability

The raw data supporting the conclusions of this article will be made available by the authors, without undue reservation.
